# Retraction: Peg-Asparaginase-Associated Pancreatitis in Chemotherapy-Treated Pediatric Patients: A 5-Year Retrospective Study

**DOI:** 10.3389/fonc.2021.709636

**Published:** 2021-06-03

**Authors:** Frontiers Editorial Office

**Affiliations:** Frontiers Media SA, Lausanne, Switzerland

The Journal and Authors retract the 28 October 2020 article cited above for the following reasons provided by the Authors:

Following publication, concerns were raised regarding data misrepresentation. The authors failed to provide a satisfactory explanation during the investigation, which was conducted in accordance with Frontiers’ policies.

This retraction was approved by the Chief Editors of Frontiers in Oncology and Chief Executive Editor. The authors agreed to this retraction.

